# Studies on the mechanical and thermal stability of *Calotropis gigantea* fibre-reinforced bran nano particulates epoxy composite

**DOI:** 10.1038/s41598-023-42316-6

**Published:** 2023-09-28

**Authors:** Thandavamoorthy Raja, Yuvarajan Devarajan, Subash Thanappan

**Affiliations:** 1 Material Science Lab, Department of Prosthodontics, Saveetha Dental College and Hospitals, SIMATS, Saveetha University, Chennai, Tamil Nadu 600077 India; 2 Department of Mechanical Engineering, Saveetha School of Engineering, SIMATS, Saveetha University, Chennai, Tamil Nadu India; 3 Department of Civil Engineering, Ambo University, Ambo, Ethiopia

**Keywords:** Engineering, Mechanical engineering

## Abstract

In recent trends, the usage of synthetic materials has been reduced by introducing natural fibres for lightweight applications. In this study, Madar (*Calotropis gigantea*) fibre is selected for the reinforcement phase (40%), and the epoxy polymer is blended with bran filler selected as a matrix material. To calculate hybrid composite mechanical characteristics, five composite laminates with different fibre/filler weight ratios were made. The results show that when the weight ratio of madar fibre increased, the superior mechanical properties were observed in the composite laminate sample (A), such as tensile strength (20.85 MPa), flexural strength (24.14 MPa), impact energy absorption (23 J) compared with an increasing the weight ratio of bran nanofiller to this composite material. At the same time, increasing bran nanofillers can improve thermal stability up to 445 °C of degrading temperature. To analyse the surface interaction between the madar fibres, bran nanofillers, and epoxy matrix by conducting the scanning electron microscope (SEM) analysis before subjecting to the mechanical test and also to identify the failure mode by conducting the SEM test after the laminates are broken during the mechanical tests of the hybrid composite.

## Introduction

Natural fibre-reinforced composites are being developed for use in the study to replace synthetic fibre-reinforced composites. The matrix and fibres have been replaced with environmentally friendly and biodegradable components^1^. Buildings, bridges, and structures such as boat hulls, swimming pool panels, racing car bodies, shower stalls, bathtubs, storage containers, imitation granite, and cultured marble basins and countertops are typically constructed from composite materials, also increasingly used in automotive applications in general ^2^. Fillers are typically comprised of fine glass, quartz, or silica and are added to improve the restoration's elastic modulus, tensile strength, hardness, and abrasion resistance and reduce polymerisation shrinkage. Interior components, such as door panels, dashboard components, parcel compartments, seat cushions, backrests, cable linings, etc., typically feature composite materials reinforced with natural fibres^3^. Due to the high demand for mechanical strength, exterior applications are confined. Natural fibre composites are durable, inexpensive, lightweight, have high specific strength, are non-abrasive, have fairly excellent mechanical properties, are environmentally benign, and biodegrade^4^. Technical hemp, jute, and flax are natural fibres with excellent mechanical, acoustic, and thermal insulation properties. The fibre content and length are the most influential factors in a natural fibre-reinforced composite's mechanical and physical properties. In recent trends, more research is being conducted on characterising natural fibres. *Calotropis gigantea* fibres can be used as reinforcement due to their cellulose content, crystallinity index (56.08%), crystallite size (2.05 nm), and thermal stability (> 220 °C), according to these values are comparable to those of other natural fibres presently used as reinforcing agents in polymers, such as *Cocos nucifera*, *Luffa cyclin-drive*, *Eucalyptus grandis*, *Pinus elliotti*, *Curaua*, etc.^5^. Its relatively large greyish-green leaves are 5–20 cm long and 4–10 cm wide and are produced in pairs. Herbaceous lower portions are woody, aerial, erect, branched, cylindrical, and solid, while upper portions are covered with woolly filaments, pale green, and contain latex^6^. In addition to a high strength-to-weight ratio, the Madar Fibre-reinforced polymer composite demonstrates extraordinary properties such as high durability, stiffness, damping properties, flexural strength, and resistance to corrosion, abrasion, impact, and fire^7^. Increases in fibre content will increase the tensile property. Before the breakdown of the polypropylene matrix, we used sugarcane fibre composition in this investigation. Sugarcane fibre, when compared to other fibre composites, will have high thermal stability at 450°C^8^. Differential scanning calorimetry (DSC) is a type of calorimetry where an increase in perceived temperature indicates that the fibre has reached the state of the nucleating site. Because the polymer crystallises, the mechanical and crystallinity properties of the material improve in composites made from sugarcane fibres. The utilisation of such strands can be defended for aviation and military applications where the significant expense of the filaments is not of high significance^9^. Reinforcement of fibre exposed by its length is much more prominent than cross-sectional measurements. At the same time, the proportion of length to the cross-sectional measurement, known as angle proportion, can fluctuate significantly. Fibre-reinforced plastics (FRP) are effectively utilised for different utilisations of the present aviation innovation as a result of their astounding explicit properties, for example, high explicit quality and solidness, low weight, and the capability of advancement by orientating (particularly persistent) fibres along with the load conduct^10^. The incorporation of flax, jute, hemp, ramie, and kenaf fibres extracted from plant stem and leaf fibres are disengaged from the plant leaves^11^. To incorporate the sisal, pineapple, and banana fibres extracted from the plant’s outer layer of bark and seed or organic product strands are separated from seed or natural products^12^. An extrusion of gas atomised control containing 62% beryllium and 38% Al was used to test the materials. The findings reveal that the mechanical and thermal properties of hybrid composites, such as fracture toughness, fatigue, thermal conductivity, and coefficient of thermal expansion, have improved. 20% natural fibre composites exhibit a 33% increase in tensile strength and a 75% increase in tensile modulus. Based on these findings, coir fibres with matrix confirmed the role of preserved coir fibre and served as a reinforcing agent rather than a filler^13^. Adjusting the surface of PALF (Pineapple leaves fibre) and KF (Kenaf fibre) for fabricating the KF/PF hybrid composites offers superior interfacial strength, enabling the materials' mechanical strength. The mechanical characteristics of fibre-strengthened composite rely upon numerous parameters, such as fibre quality, modulus, fibre length, and orientation, notwithstanding the fibre-network interfacial bond quality^14^. Fibre-reinforced polymer matrix becomes a significant consideration in various applications as a result of the great properties and better points of interest of natural fibre over synthetic fibres in terms of its moderately low weight, less harm to handling devices, great relative mechanical properties, for example, tensile modulus and flexural modulus, improved surface completion of shaped parts composite, sustainable resources, being plenteous, adaptability during preparing, biodegradability, and negligible wellbeing dangers^15^. The wide utilisation of NFRPC (Natural fibre-reinforced polymer composite) is developing quickly in various design fields. An increase in the 9 per cent chopped madar fibre weight fraction increases the hybrid composite's impact energy absorption capability more considerably than in the banyan fibre weight fraction. The dominant mode was discovered via scanning electron microscope surface morphological analysis. There is a risk of failure. The mechanical properties of bidirectional madar fibre epoxy composites prepared by the hand lay-up technique, such as hardness, tensile strength, and impact strength, increased with the weight of Madar^16^. The flexural and interlaminar strengths initially diminished up to 12 wt% madar fibre loading and then increased to 48 wt% madar fibre loading. The decrease in cavities caused by an increase in madar fibre input in composites is one of the reasons the mechanical properties of bidirectional Madar/epoxy composites increase^17^. The tensile strength and elastic modulus of kenaf blended with polyester were 381–712 MPa and 27 GPa, respectively^18^. In another study, the mechanical properties of madar and banana fibre-reinforced epoxy hybrid composites were investigated. The study found that adding banana to madar/epoxy composites increased the mechanical properties such as tensile, flexural, and impact strengths by 16%, 3.9%, and 31.4%, respectively^19^. The various types of natural fibre-reinforced polymer composite have gotten a strange significance in various car applications by numerous car organisations, such as German auto organisations, such as Audi Group, Ford, Volkswagen, Mercedes, etc. Various types of natural fibres are replacing synthetic fibre usages in the composite. Also, the thermal degradation of composite needs to identify the efficiency of natural fibre composite^20^.

The above motivational research work has started to develop a composite laminate by using chopped madar fibre, bran nanofillers cellulose and epoxy matrix varying with weight fractions of madar fibre and nanofillers to quantify the mechanical effect and thermal stability of hybrid composite and the surface morphology of hybrid composites that can identify with SEM analysis.

## Materials and experimental process

**Figure 1:**
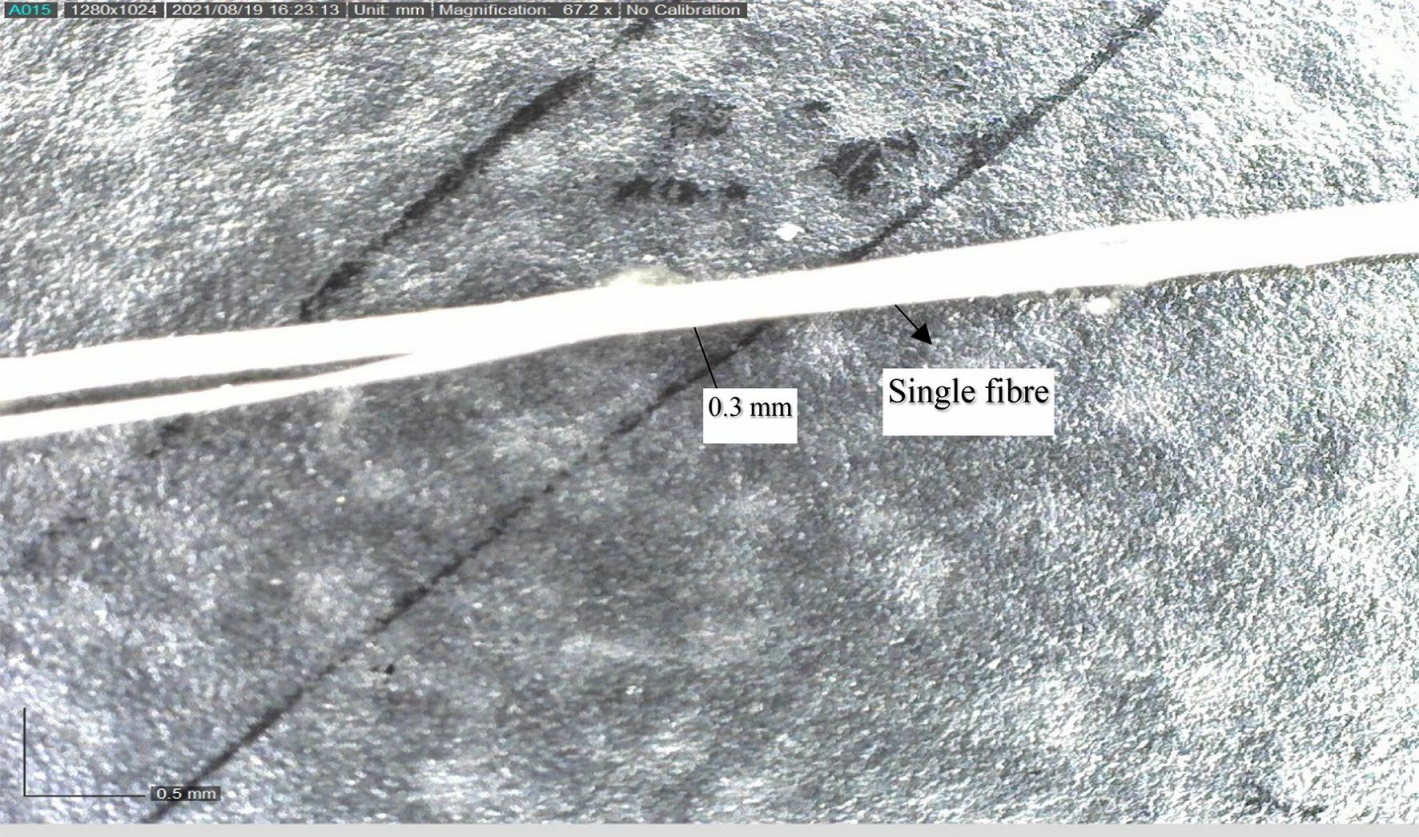
Microscopic image of a single madar fibre.

This study complies with relevant institutional, national, and international guidelines and legislation. This work aims to create a composite laminate utilising natural fibre, filler, and an epoxy matrix. The matrix comprises Araldite (LY 556), Bisphenol-F type epoxy polymer resin from Huntsman, and Araldite HY 951 hardner from Araldite, Javanthi enterpriser, Chennai, India. Go Green Pvt supplied the Madar fibre and bran nanofiller. Limited, Chennai, India. Due to the shortage in the length of madar twigs, the fibre can be utilised as a chopped type for reinforcement material. The water retting process was used to extract the madar fibre, which means water can enter the stem's central part, swelling the internal cells and causing the plants' outer covering to break^21^. The presence of bacteria and moisture in plants causes vast sections of cellular tissues and sticky compounds that wrap fibres to be broken down, separating individual fibres from the plant, and madar fibre has been censored to chopped type for fabrication of composite^22^. The general properties of madar fibres are the tensile strength is 5.8 MPa, Young’s modulus is 1.3 GPa, and density is 1.67 g/cm^3^and to expand the characteristics of the hybrid composite, bran nanofillers with a particle size of 100 μm (average) and density is 0.25 g/cm^3^ utilised as a filler ingredient^23^. The hybrid composites were fabricated via a traditional hand lay-up technique, with reinforcement, filler, and matrix hybridisation employed to construct the composite. For the fabrication procedure, a mild stainless steel mould was utilised initially to apply liquid wax as a mould release agent on a 25 × 25 cm steel mould, predefined 60% epoxy resin was mixed with hardener at a 10:1 ratio, and 40% reinforcement of chopped madar fibres and bran nanofillers was taken, the matrix was fixed with 60% for all five samples, and the chopped madar fibres, bran nanofillers are varied with five different weight fractions in grams are followed 140/10, 130/20, 120/30, 110/40, 100/50 of hybrid composite. On the steel box, liquid wax was applied as a mould release agent, followed by a layer of minced madar fibre and an epoxy matrix with a bran nano-infill blend to the required thickness of 5–8 mm^24^. The process was repeated with five different weight fractions of madar fibre and nanofillers. After completion of fabrication, the laminates were cured in a hot furnace at a rate of 50 °C per minute for two hours and compressed with five kilograms per square centimetre of mould area for twenty-four hours to improve the curing of the hybrid composite. The source materials of single madar fibre in a microscopic image, in Fig. 1

**Figure 2:**
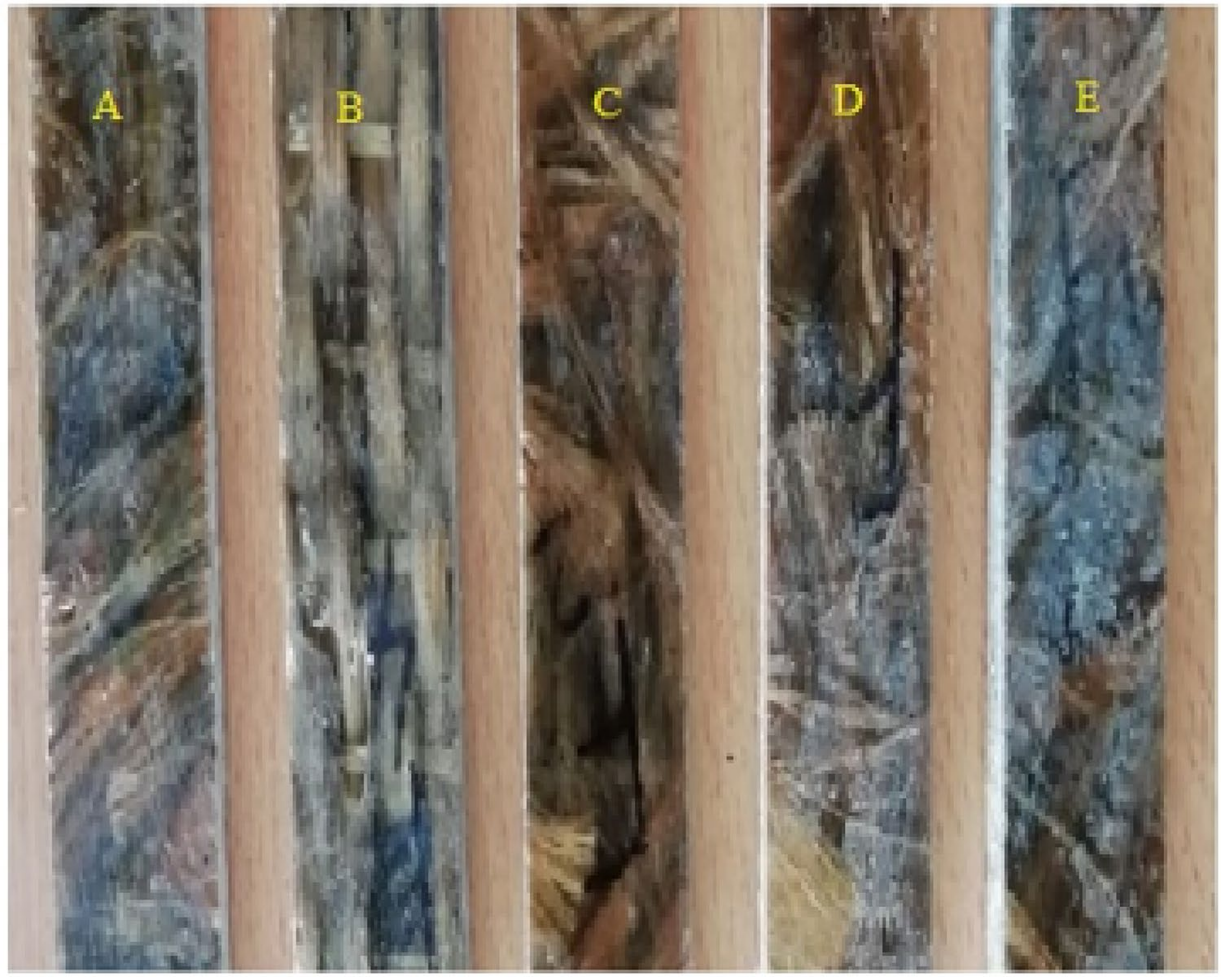
Natural fibre composite laminate testing samples.

The method was repeated for all samples, and a specimen was collected from each sample following ASTM standards to conduct various analyses. The weight ratio of chopped madar fibre, nanofiller, and epoxy polymer matrix materials are tabulated in Table 1. The hybrid composite was fabricated with chopped madar fibre and added bran nanofiller epoxy polymer and the samples are conducting mechanical testing such as tensile test, flexural test, and impact energy absorption, also conducting the Thermogravimetric analysis. The tensile test was conducted on Universal Testing Machine (TINIUS OLSEN H10KT) with a slow loading rate of 1 mm/min by 10 KN mode of an experiment as per ASTM D638 standard 165 × 13 × 3.2 mm dimension was taken, position measurement accuracy was observed + /− 0.01% of reading or 0.001 mm, and load accuracy of 0.5% of the specified load is achieved. The flexural test was conducted in 3 points bending mode of ASTM D790 standard with the dimensions of 125 × 12.7 × 3.2 mm, force ratio of 100:1 (i.e. the load cell to 1.0% of capacity with no loss of accuracy) and for impact strength by conducting Izod impact test, ASTM D256 standard with 65 × 12.7 × 3.2 mm dimensions as followed and for thermal stability by conducting the Thermogravimetric analysis ASTM E1131 standard was followed^25^. Using a 115 EVO 18 SEM, the surface interaction and morphology of the hybrid composite are investigated (Carl Zeiss, Oberkochen, Germany). To improve visibility, the hybrid composites are 116 sputter coated with gold particles before surface microanalysis. The specimens for conducting all tests per the ASTM standard and three specimens were taken from all samples for each analysis. They revealed the average properties of this hybrid composite. The tested samples are shown in Fig. 2.

**Table 1:** The weight ratio of natural fibre composite laminates.

Sample	Weight of madar fibre in g	The volume of madar fibre in mm^3^	Weight of bran nanofiller in g	The volume of bran nanofiller in mm^3^	Weight of epoxy matrix in g	Volume of epoxy matrix in mm^3^	Weight of composite laminate in g
A	140	83.33	10	40	220	157	370
B	130	77.84	20	80	220	157	370
C	120	71.85	30	120	220	157	370
D	110	65.86	40	160	220	157	370
E	100	59.88	50	200	220	157	370

## Results and discussion

**Figure 3:**
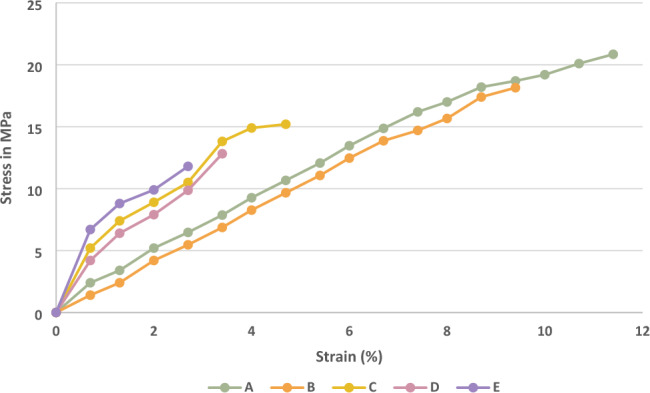
Stress versus strain curve of hybrid composite.

**Figure 4:**
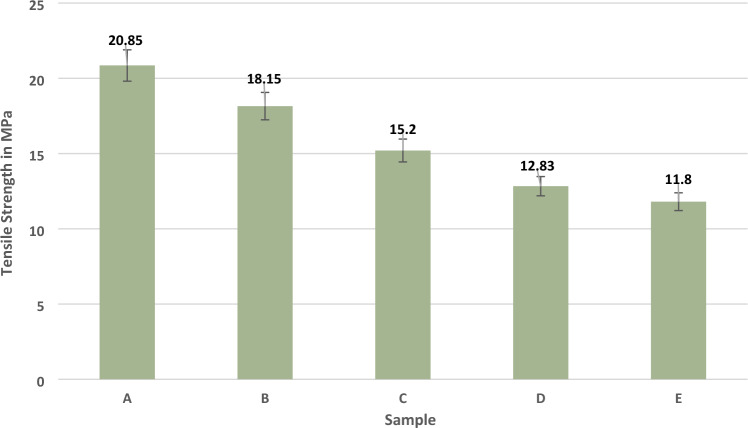
Tensile strength of composite laminates.

In this research, mechanical testing of hybrid composites has been conducted per ASTM standards. The results of tensile, flexural, and impact strength show the significant response of hybrid composites. The stress vs strain curve of composite laminates is shown in Fig. 3. These results can reveal the elastic capacity of chopped madar fibre composite. Sample A's maximum stress and strain values are 20.85 MPa and 11.4%. When the increasing stress can give a similar impact increasing the strain value of hybrid composite to reach the maximum elastic capacity, the material is moved to the breaking point and the same results were repeated in all the other samples when the stress increased parallelly strain value was increased upto the elastic limit of the hybrid composite. The volume of madar fibre is more in samples A and B. Therefore, madar fibre can give more stress during the tensile loading compared to bran nanofiller in the hybrid composite. The tensile strength of the hybrid composite is given in Fig. 4. Coefficient variation for this hybrid composite during the mechanical properties has been calculated by,

$${\text{Coefficient}}\;{\text{of}}\;{\text{variation}} = {\text{Standard}}\;{\text{deviation}}/{\text{Mean}}$$

The Madar fibre composite stability in tensile strength is given in Table 2. The above results are revealed when the increase of fibre reinforcement weight fraction was given a positive influence compared to the increase of filler percentage in the composite laminates. The hybrid composite comprises chopped madar fibre, bran nanofiller, and epoxy matrix. Sample A’s tensile strength of 20.85 MPa, is the maximum value compared with other samples. It contains 140 g chopped madar fibre and 10 g bran nanofiller, is indicated the maximum strength is dependent on fibre reinforcement, and sample B is a 12% loss of tensile strength compared with sample A and at the same time when the increase of fibre ratio can reveal the increase in tensile strength of composite laminates. In sample C, the increase of 30 g filler can show a negative influence of 25% compared with sample A. In the sample, E was given a minor tensile strength of 11.8 MPa, 45% lesser than sample A. In another work, flax fibre composite was given 42 MPa of tensile strength with the usage of woven flax mat, due to the load transfer from one to another point can uniformly distribute in mat form reinforcement, and it was less in short fibres used as reinforcement was identified^26^. Similar research was developed using basalt/flax fibres hybrid composite. The samples exhibited the highest tensile strength owing to a basalt fibre layer. A single flax fibre layer has lower tensile strength than other hybrid composite samples. If the composite's tensile strength decreases while the cross-linking rate increases, post-curing has altered one or both components^27^. Therefore, when the gradual axial pulling load is applied on the hybrid composite laminates, it can reveal that reinforcement plays a significant role when fabricating natural composite compared with other filler materials. In each sample, five different specimens were taken for different trials, and the error bar indicates the average values from five different trials of the experiments of tensile, flexural, and impact analysis of composite laminates. The material stability in the flexural test is given in Table 3.

**Table 2:** Material stability in tensile strength of hybrid composite.

Sample	Standard deviation	Mean	Coefficient of variation
A	0.24	20.85	0.011
B	0.20	18.15	0.011
C	0.39	15.20	0.025
D	0.17	12.83	0.013
E	0.41	11.90	0.034

**Table 3:** Material stability in flexural strength of hybrid composite.

Sample	Standard deviation	Mean	Coefficient of variation
A	0.15	24.14	0.006
B	0.16	23.29	0.006
C	0.14	19.50	0.007
D	0.24	17.67	0.013
E	0.37	13.00	0.028

**Figure 5:**
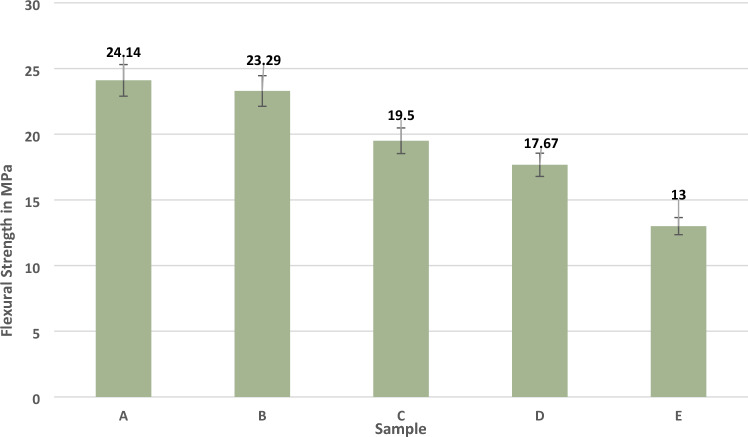
Flexural strength of composite laminates.

The flexural test experiment can reveal the load withstood during the bending load condition in the 3 points bending test. During this experiment, the 2 mm deflection was calculated in the hybrid composite^28^. The flexural strengths of the composite laminates are given in Fig. 5. Sample, A is the significant result among all other samples, and it was given 24.14 MPa flexural strength. This result is 3% more compared to sample B, and the minor flexural strength identified in sample E contained maximum bran nanofiller material and a minimum of chopped madar fibre. Therefore, increasing chopped madar fibre material can improve the bending load in the flexural strength of hybrid composite compared with increasing nanofillers into the composite laminates. In another work, increasing sisal fibre can improve the higher flexural energy compared to the sawdust cellulose content present in the composite^29^. The similar outcome revealed in this work being improved with fibre reinforcement can give significant outcomes^30^. In another research work, the composite laminates containing less flax have a minimum flexural strength of 32 MPa, 59.4% less than basalt fibre bending capacity. During bending load, basalt fibres can transmit the load in a different direction and increase bending capacity due to incorporating a thicker basalt layer into composite samples. In contrast, flax fibre does not possess a greater bending capacity than basalt fibre^31^. In sample C, 19.5 MPa were given, and the flexural strength showing the equal ratio of chopped madar fibre and bran nanofillers can reduce the flexural loss compared to sample D and sample E.

**Figure 6:**
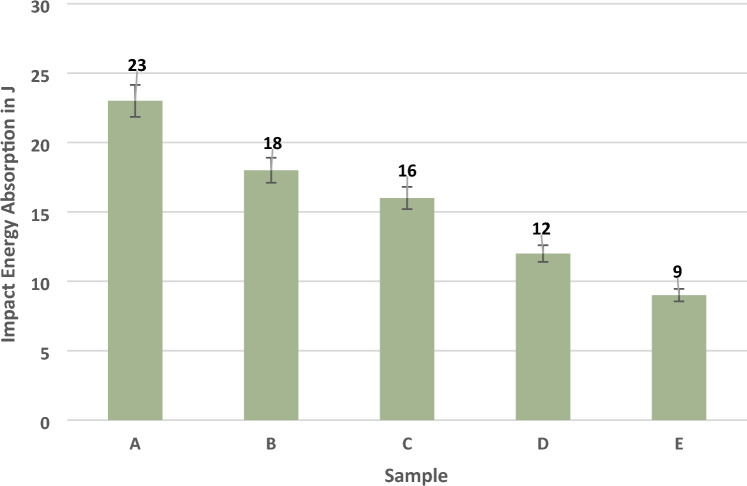
Impact Energy of composite laminates.

The impact energy coefficient is given in Table 4. This work analyses the impact energy absorption capacity by conducting the Izod impact strength on this composite laminate. The results are the same as tensile and flexural strength. The chopped madar fibre can resist more impact load because sample A absorbs more energy of 23 J and sample E absorbs minimum energy of 9 J, indicating that the nanofiller of bran nanofillers can break easily when applied suddenly. At the same time, the fibre material can combine formally and resist the impact load more, absorbing the energy in sample B at 18 J. This result is 21% less than sample A. It indicates that increasing 10 g can have more impact on the hybrid composite during the sudden force applied to this composite. Similar work was done with a natural fibre of basalt/kenaf fibre composite showing the impact energy of 19 J, due to the combination in mat form of flax/glass can withstand more load is gradually applied. It was less in sudden load because when the impact load can quickly transfer from one point to another in short fibre, it was diluted in mat form of composite^32^. The impact energies of natural composite laminates are shown in Fig. 6. The significant limitations are the usage of chopped madar fibre, which can transfer the load in the short term. The fibres cannot withstand more loads during mechanical testing, and filler materials can only improve thermal degradation. At the same time, the mat form of reinforcement can give the potential output in the hybrid composite's mechanical and thermal behaviour.

**Table 4:** Material stability in impact energy of the hybrid composite.

Sample	Standard deviation	Mean	Coefficient of variation
A	0.41	23	0.017
B	0.17	18	0.009
C	0.14	16	0.008
D	0.29	12	0.024
E	0.13	9	0.014

The effect of fibre content (20–50 wt%) on abaca-reinforced polypropylene (PP) composite is compared to jute/flax-reinforced polypropylene composite under impact test. Results revealed the impact strength increased from 20 to 40 wt% fibre content. Still, the impact strength starts to decrease when adding 40–50 wt% fibre content because the fibre/matrix ratio is a crucial factor in composite materials' ability to resist the impact load^33^. Analysed injection-moulded polylactide composites (PLA) and polypropylene (PP) reinforced with synthetic kevlar fibre and abaca fibres. All composites were produced with a fibre content of 30 wt%. Concerning the fibre weight, PP-based composites were evaluated with a 5 wt% maleated polypropylene (MAPP) content. At room temperature, the NI efficacy of PP-based materials was substantially higher than that of PLA-based materials, whereas, at 30 °C, the opposite was true. The outcomes demonstrated a distinct distinction between composites reinforced with abaca fibre and synthetic cellulose^34^. Usually, natural fibres are more moisture content, and the presence of lignin and hemicellulose is the primary reason for less bonding capacity with polymer matrix. To avoid this, fibre treatment can improve 2% more mechanical properties of natural fibre composite.

**Figure 7:**
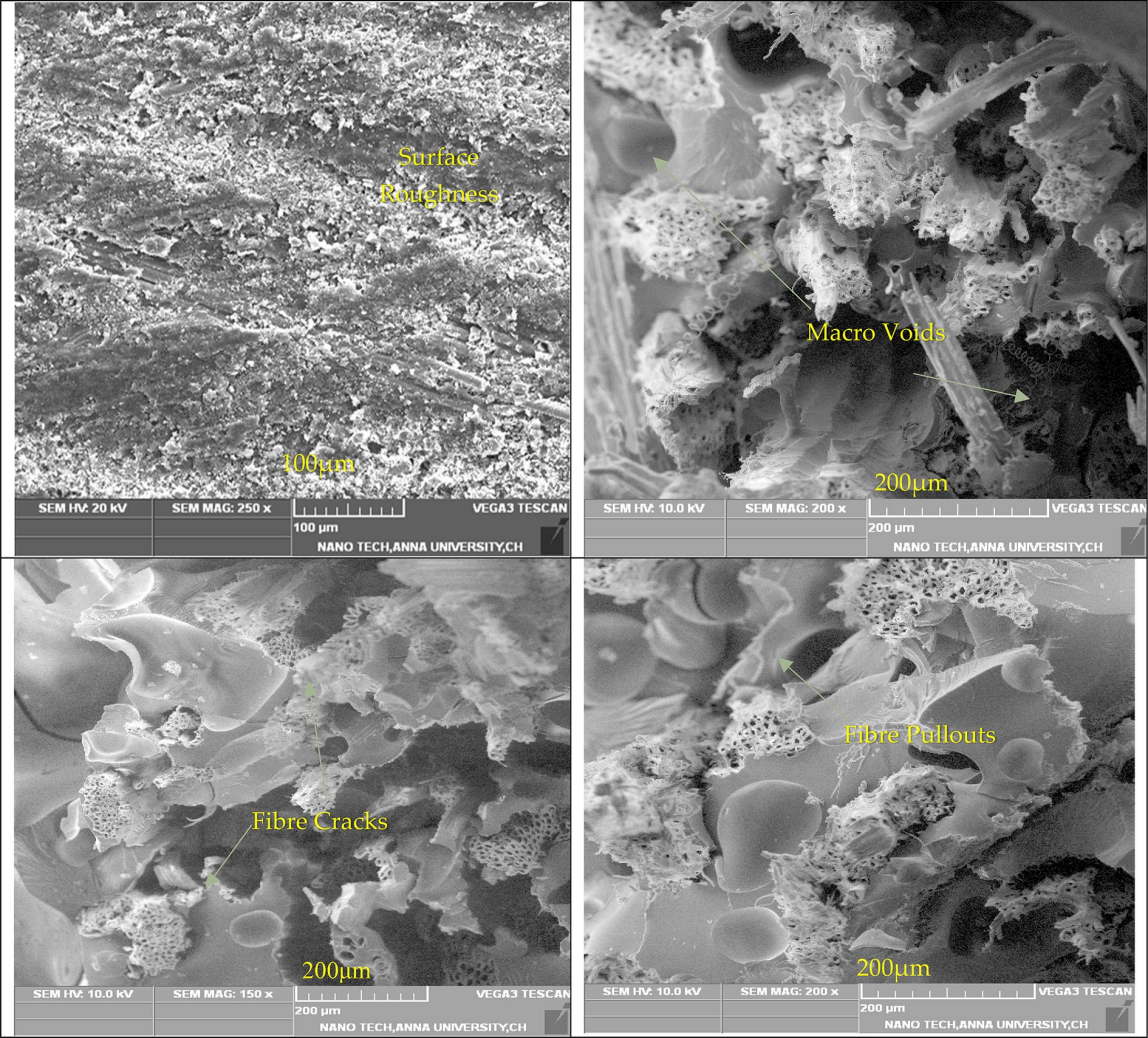
SEM image of composite laminates.

The above SEM micrographs revealed before and after the fracture of composite laminates, during the fabrication process, the epoxy resin can improve the bubbling and porosity due to atmospheric conditions. It can affect the surface finishing of composite laminates, and the failure mode can be identified from this micrograph^35^. The primary reason is that the stress concentration factor is more due to a short form of fibre. The cracks and fibre pullouts are revealed as the higher stress concentration factor presents in laminates and voids and fibre cracks in the composite laminates. Alkenes, phenolic hydroxyl groups, aromatic groups, and various functional groups with oxygen are the main components^34^. The SEM image of the composite laminates is shown in Fig. 7. The bonding between fibre and matrix is improved, and at the same time, the bonding between fibre and filler gets weakened in the natural composite laminates.

### Thermal stability of madar fibre composite laminates

**Figure 8:**
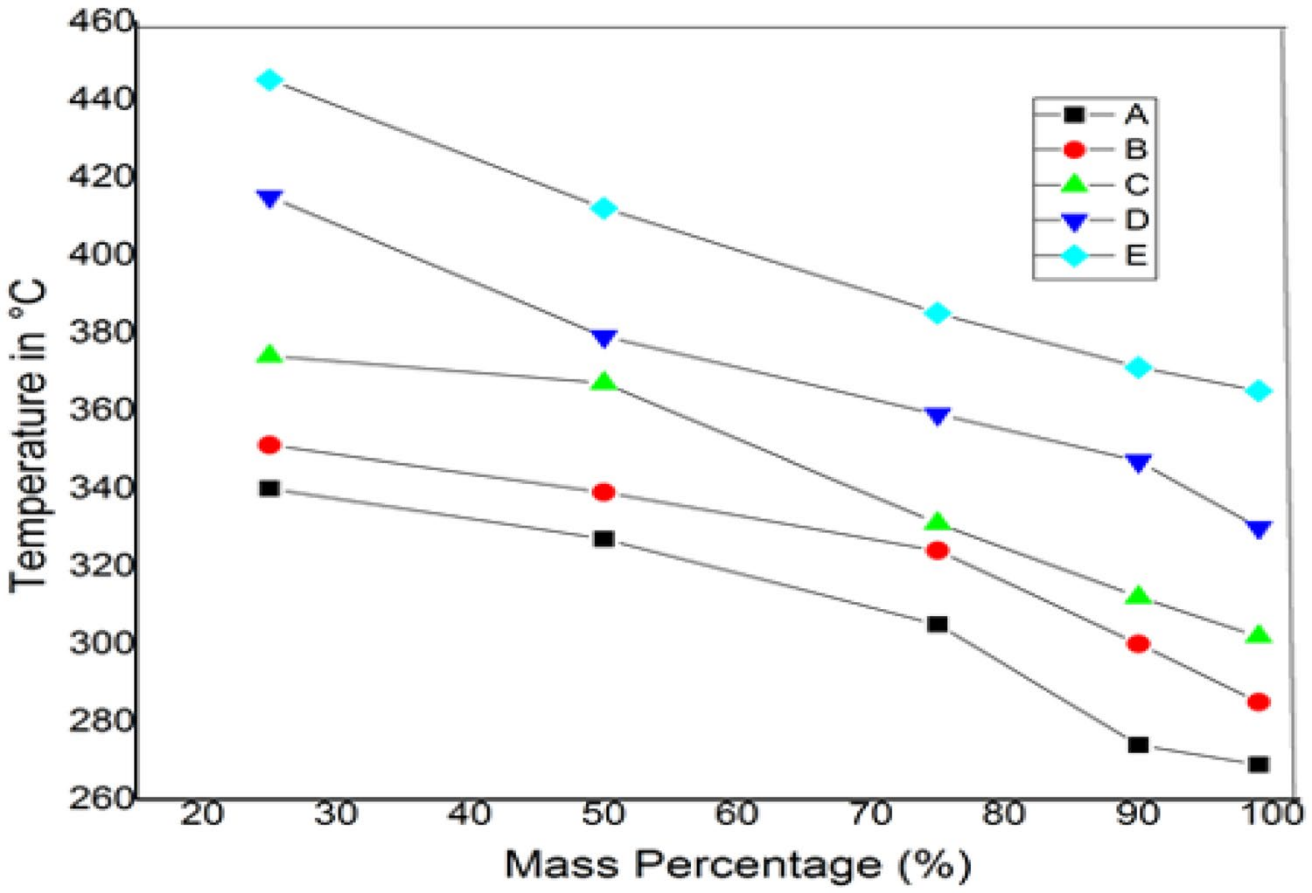
The graph between mass percentage versus temperature of hybrid composite.

**Figure 9:**
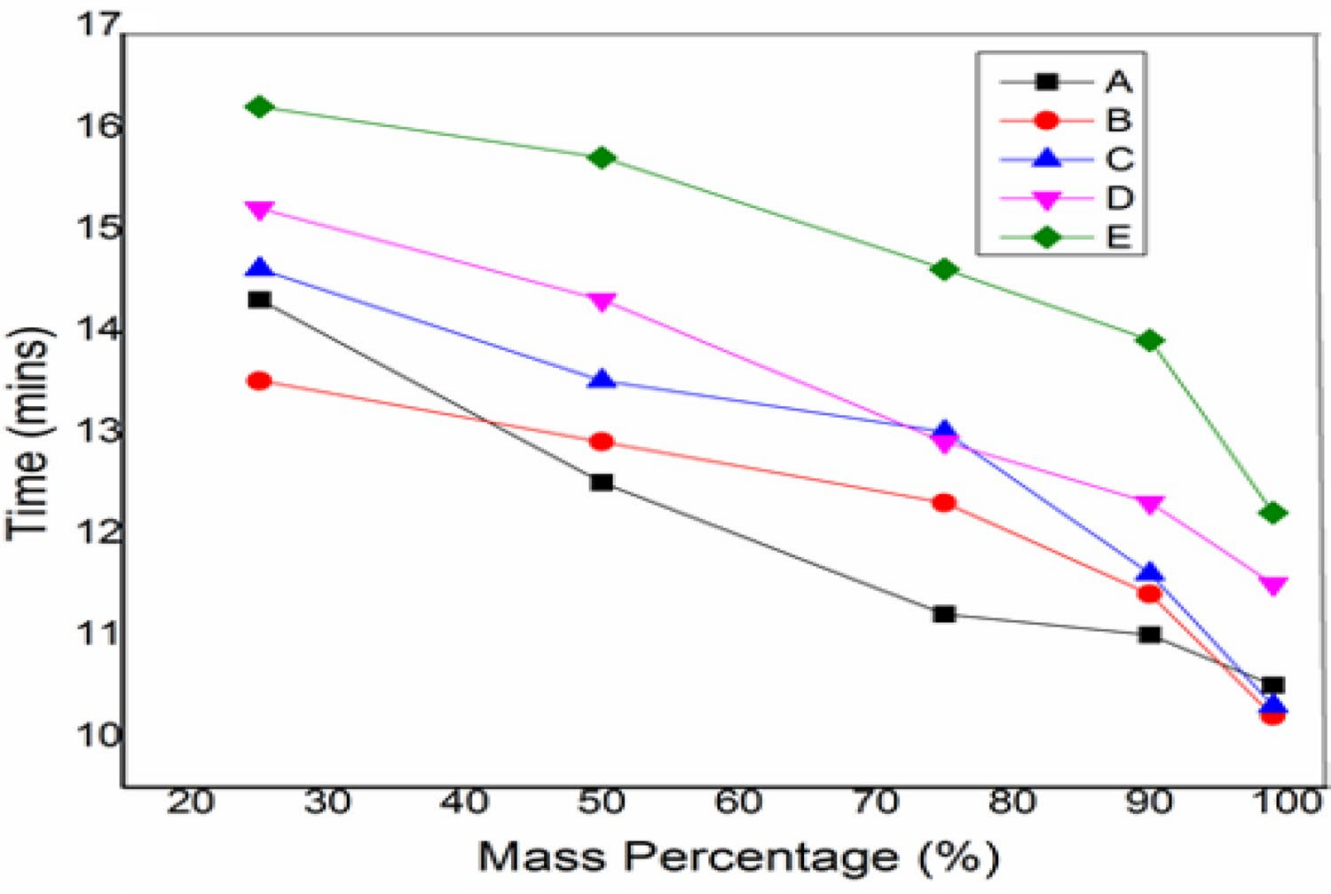
The graph between mass percentage versus time of the hybrid composite.

TGA tests were performed on the proposed madar fibre-reinforced hybrid composite to determine the thermal stability of five different weight fractions and the material degradation of this composite. This study is used to determine the stability of the material under various temperature increases and the time it takes for the substance to degrade^35^. The initial temperature was taken for the material mass of 15–14.9 g at 365 °C, and the time taken during the operation was 12.5 min in this hybrid composite sample E. In comparison, the initial temperature was taken for the material mass of 15–14.9 g at 269 °C, and the time taken during the operation was 10.5 min in sample A. In another research, the TGA analysis of neem twig fibre revealed that its thermal stability is superior to other natural fibres such as sisal and banana. The solid stage began at an ambient temperature of 200 °C, the glass transition temperature between 210 and 350 °C, and the elastic region began at 352 °C^36^. This thermogravimetric analysis shows that growing natural bran nanofillers can resist greater temperatures at 445 °C of working temperature with a corresponding mass of 25% of the initial mass. The material can degrade once it reaches a maximum temperature of 445 °C. Figures 8 and 9 show the graph of mass percentage with temperature and mass% with the time taken for mass loss at different percentages of hybrid composites, revealing the thermal stability of chopped madar fibres blended with bran nanofillers hybrid polymer composites.

## Conclusion

The composite laminates were developed with chopped madar fibre reinforced with bran filler blended epoxy matrix. The mechanical and thermogravimetric experiment was conducted to analyse the general properties of mechanical and temperature variation of composite laminates. The significant findings taken from this research work have been as follows. In tensile strength, flexural strength and impact energy was improved by an average of 60% due to chopped madar fibre supply compared with bran nanofillers. It can reveal that the addition of bran nanofiller negatively influenced the natural composite's mechanical properties and that supplying chopped madar fibre reinforcement can improve the mechanical properties of the madar fibre composite. Based on the outcome, the madar fibre can be used as reinforcement in polymer composite for lightweight structural applications due to the significant results observed in the sample (A) tensile strength (20.85 MPa), flexural strength (24.1 MPa), impact energy (23 J) among other four samples weight fraction. In thermal stability, supplying bran nanofiller improved thermal stability when compared to increasing madar fibre with an average of 24%. Superior thermal stability was observed in sample E with a maximum degradation temperature of 445 °C. The time taken for the material to degrade, 16.2 min, is required to reach the sample mass of 25% of this hybrid composite. Therefore, adding bran nanofillers into the natural composite is highly suitable for preparing the thermal insulation materials of hybrid composites.

## Data Availability

The datasets used and/or analysed during the current study available from the corresponding author on reasonable request.
